# Development and acceptability of a cereal bar with *Atta sexdens* ant flour

**DOI:** 10.12688/f1000research.135516.1

**Published:** 2023-07-19

**Authors:** Michelle Lozada-Urbano, Jessica Bendezú Ccanto, Julissa Condori Chura, Oriana Rivera-Lozada, Jaime A. Yañez

**Affiliations:** 1 Centro de Investigación en Biodiversidad para la Salud, Universidad Norbert Wiener, Lima District, Lima Region, Peru; 2 South American Center for Education and Research in Public Health, Universidad Norbert Wiener, Lima District, Lima Region, Peru; 3 Dresden Food Ingredients S.A., Lima, Peru; 4 Medicina Humana, Universidad Privada San Juan Bautista, Lima District, Lima Region, Peru

**Keywords:** Entomophagy; energy bar; Atta sexdens; protein; ant; insect edible.

## Abstract

In Peru, insect consumption, as a nutritional complement or as the main source in the diet, is limited to the regions of our the central jungle where *Atta sexdens* ants are consumed. An energy bar based on Andean grains with *Atta sexdens* ant flour was formulated. The ants were obtained from the department of San Martin, district of Rioja, province of Rioja. Four different formulations were prepared with different *Atta sexdens* ant flour concentrations: 13%, 17%, 20%, and 23%. Moisture, total fat, ash, proteins, fiber, carbohydrates, instrumental texture, and organoleptic analysis (taste, texture, and color) were performed. The developed cereal energy bar presented a 10 g protein content in 100 g of the final product. Acceptability was evaluated in adolescents and young adult populations. The developed cereal bars presented a high protein content, adequate organoleptic properties and high acceptability.

## Introduction

The Food and Agriculture Organization of the United Nations (FAO) recommends the consumption of edible insects because It is a sustainable alternative that replaces animal meat, such as beef, poultry, and fish. ^1^ In Africa, Latin America, and Asia, these insects are commonly used to feed their populations. ^2^ In Mexico alone, 549 species of ants have been reported to be consumed by diverse ethnic groups of this country. ^3^

It has been reported that in Thailand their diet has been diversified with the consumption of sago worms ^4^ and *Acheta domesticus* grasshoppers. ^5^ Similarly, ants and bees have been reported to be consumed in Cameroon. ^6^ In Peru, the consumption of Suri, a species of *Rhynchophorus palmarum* worm, and ants known as Mamako or Siqui Sapa (Atta sp.) has been reported to be widely consumed because of their pleasant taste. ^7^

Insects have high nutritional value because of their high content of fats, proteins, vitamins, fiber and minerals, which is a great opportunity for the development of healthy foods. ^8^ Mealworm has a high content of unsaturated omega-3 fatty acids and healthy fats in similar quantities than fish, and their content of protein, vitamin and micronutrients is similar to fish and meat. ^9^ In addition, protein from insects has been reported to produce a lower impact in the environment than obtaining protein from cattle or poultry. ^2^

Many insect species are consumed alone or have been included in other preparations. In the Netherlands, pasta has been made with durum wheat with the addition of cricket powder. ^10^ Cereal bars are In this study, an accessible and an easily consumed product by adolescents and children. Consuming them in the morning and mid-morning can be beneficial to humor and memory behavioral aspects. ^11^ According to the *Codex Alimentarius*, a cereal bar is mainly prepared with one or more ground cereals. ^12^ Sales of nutrition bars increased almost tenfold in the last decade, and may be a way to utilize processed insect flour, ^13^ because of the easiness of consumption. To the best of our knowledge there are no studies that have included ant flour in cereal bars. Therefore, the objective of this study was to develop a cereal bar with Andean cereals and the addition of ant flour, evaluate its nutritional value, fracturability, compression. A microbiological analysis was performed, as well as acceptability in a population of adolescents and young people.

## Methods

### Obtaining Atta sexdens ants (Siqui sapa) and Ant flour preparation

*Atta sexdens* ants have been taxonomically assessed based on their morphological characteristics. ^14^ These ants are brown or dark brown in color and the adult ants present three pairs of spines of approximately 6 to 14 mm, build their nests in underground tunnels of up to eight meters deep and can cover an area of 50 to 100 m ^2^, and cut pieces of leaves, which they transport to their nest following visible paths. ^15^

The siqui sapa ants were brought from the department of San Martín, province and district of Rioja, and were obtained through a distributor. The ants were transported to Lima and delivered in vacuum packaging to maximize the conservation of the product.

For the preparation of ant flour, the ants were placed in an oven at 65°C for 220 minutes. Of a sample of 100 g, we obtained 72.1 g of our final product. This was taken to an electric grinder until the flour was obtained. To avoid lumps and large particles, the flour passed through a stainless-steel sieve to obtain a homogeneous product.

### Materials for the preparation of the energy bar

The ingredients used were expanded kiwicha (100 g: 24.6 g protein, 7 g carbohydrates) and quinoa (100 g, 8 g protein, 85 g carbohydrates), also called expanded cereals. The dried fruits used were pecans (100 g: 9.1 g protein, 73.8 g fat, 11.7 g carbohydrate), peanuts (100 g: 27.1 g protein, 51 g fat, 16 g carbohydrate), almonds (100 g: 23.4 g protein, 54.1 g fat, 14.3 g carbohydrate), raisins (100 g: 2.4 g protein, 63.8 g carbohydrates), and coconut (100 g: 12 g de protein, 23 g total fat, 26 g carbohydrates). Shredded coconut and unflavored gelatin were purchased from a supermarket in the city of Lima. The process began with the preparation of inverted sugar syrup at 60° Brix, H _2_O/Sugar (50%-50%), which was used to compact the energy bars.

**Figure 1.**
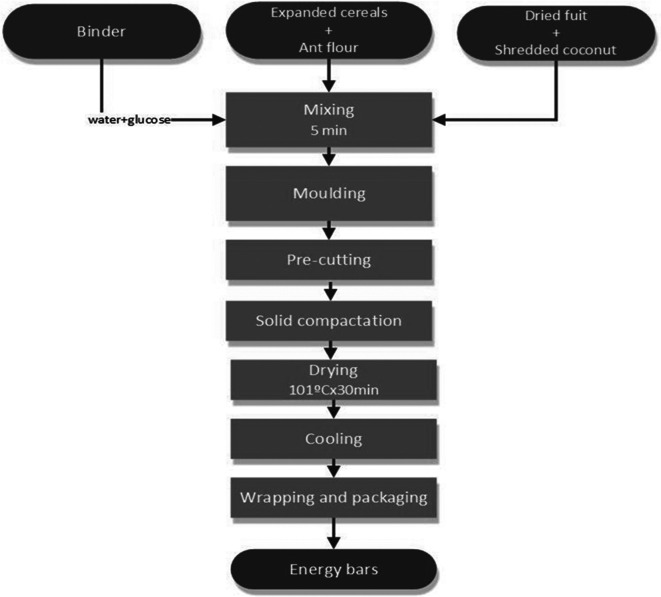
Ceral bar production process.

The expanded quinoa, kiwicha, dried fruits and ant flour were mixed together with the inverted syrup and glucose. The product was compacted with the help of molds to obtain cylindrical shapes. Figure 1 shows the procedure for the cereal bar formulation.

### Nutritional composition and texture evaluation of the final product

We measured the nutritional composition (fat, moisture, protein, carbohydrates, ash) in the four types of energy bars with ant flour in different concentrations and in the control bar, determined by AOAC standard methods. Moisture was determined according to AOAC 925.09, ^16^ fat according to AOAC 922.06, ^17^ ash according to AOAC 923.03, ^18^ protein according to AOAC 984.13 ^19^ using 6.25 as the nitrogen to protein conversion factor for all samples. Carbohydrates were calculated by subtracting the percentages of fat, moisture, protein, carbohydrates, and ash from 100%. ^20^

Two sensory quality attributes of the energy bars were evaluated. The analysis was performed using the TVT 6700 texture analyzer - Perten Instruments and TexCalc software. Fracturability is defined as the force required to fracture the sample significantly. ^21^ The three-point bending method was used with the following parameters: initial speed of 1 mm/s, speed of 0.5 mm/s during the test, 0.1961 N tensile strength, 40 mm distance between support points. ^22^ For the initial speed of compression test we used 2 mm/s; speed during the test, 2 mm/s; recoil speed, 10 mm/s; tensile strength, 0.049 N. The values of the strength required (N) to compress the specimen to 20% of its original thickness were recorded. ^23^

### Microbiological analysis

The detection method for *Salmonella* was ICMSF (International Commission Microbiological Specifications for Foods). For *Escherichia coli* the ICMSF for the determination of fecal coliform organisms was used. ^24^ For yeast ^25^ and mold ^26^ count the ICMSF method was used through seeding plates. Mesophilic aerobic microorganisms were counted using the ICMSF methods. ^27^

### Organoleptic acceptability of samples

The study was conducted by two nutritionists. A total of 100 participants were recruited, including adolescents and young people from two secondary schools, who were chosen as a population that frequently consumes bars. They received an invitation via whatsApp to participate in this study. Few participants (n=13) were contacted by calling them on their cell phones by the nutritionist in charge of administering the taste test, defined as a preference test. The inclusion criteria were adolescents and young adults who agreed to try the energy bars.

The acceptability was determined using a hedonic test, in which the recruited population was not trained. The samples had different three-digit random codes for each type of bar, a bottle of water was previously given to each participant in order to drink after each test, and strict cleanliness conditions were maintained at all times.

The report of the hedonic test results was recorded in an Excel table, which was subsequently analyzed using the SPSS statistical package. The results of the physical organoleptic analysis, the proximate analysis, and the microbiological analysis were shown in tables, indicating frequencies and percentages. The data was expressed in percentages and the experimental data was analyzed using the Kruskal-Wallis test. Normality was previously measured with Kolmogorov-Smirnov. The IBM SPSS Statistics version 25 statistical package was used.

### Ethical aspects

All participants signed the informed consent form, before participating, all participants were informed that the cereal bar was made with Andean cereals and ant flour. The study received the approval of the ethics committee Exp. N°054-2020 of the Universidad Norbert Wiener.

## Results

### Energy bar formulation

The composition of the four types of energy bars and the amount of ant flour are shown in Table 1. The data are expressed in 100 g of product.

**Table 1.** Composition of the four energy bar formulations.

Ingredients	F1 (%)	F2 (%)	F3 (%)	F4 (%)
Expanded Kiwicha	43	42	40	38
Expanded Quinoa	26	25	24	23
Shredded coconut	2	2	2	2
Dried fruit				
Raisins	3.25	3.25	3	3
Almonds	3.25	3.25	3	3
Pecans	3.25	3.25	3	3
Roasted peanuts	3.25	3.25	3	3
Unflavored gelatin	1	1	1	1
Glucose	2	2	2	2
Ant flour	13	17	20	23

### Evaluation of nutritional composition

The proximate analysis showed the increase in fat and proteins as the percentage of ant flour increases in the four different concentrations ( Table 2).

**Table 2.** Comparison among the proximate analysis of the four preparations of energy bars and a bar without ant flour (%).

	Energy bars with different concentrations				
	F0	F1	F2	F3	F4
Fat	5.58	8.61	8.49	6.27	9.62
Moisture	17.60	19.29	19.39	18.89	18
Protein (Nx6.25)	8.68	8.06	8.15	10.04	10.5
Carbohydrates	66.28	62.64	62.45	62.99	60.65
Ash	1.86	1.4	1.52	1.81	1.23

Fo: sample without ant flour; F1(13%); F2(17%); F3(20%); F4(23%).

### Evaluation of fracturability and compressibility

**Figure 2.**
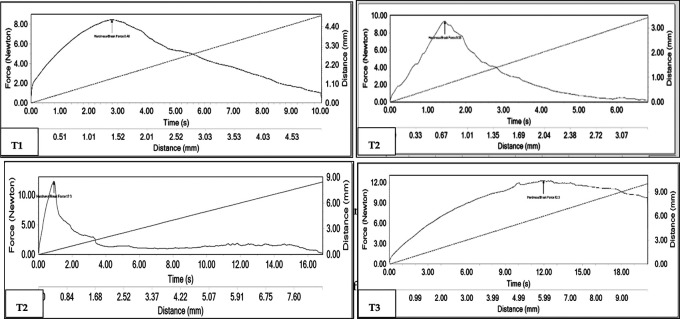
Fracturability curve in the four formulations of the protein.

The analysis of compressibility assessed hardness. The method fitted the consistency of the sample due to the fact that, in order to delimit the assays’ parameters, the greatest force exerted was within 20% of compression, and values between 80.5 N and 117.6 N were obtained ( Table 3, Figure 2).

**Table 3.** Fracturability and compressibility analysis of the four preparations of the energy bar.

Treatment	Fracturability (N)	Compressibility (N)
F1	8.48	98.9
F2	9.33	91.9
F3	12.33	80.5
F4	12.33	117.6

**Figure 3.**
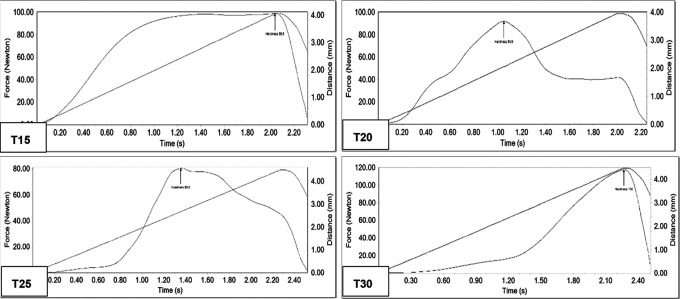
Compressibility curve in the four formulations of the protein.

Figure 2 shows the fracture curves of the treatments with 15%, 20%, 25% and 30% of ant meal T15, T20, T25 and T30 respectively. In general we can observe 3 axes that are force (N), time (s) and distance (mm). The arrow of the indicated point (Hardness) expresses the maximum force exerted to fracture the samples, for example in graph T15, the maximum force exerted to fracture the sample is observed 98.9 N, with a time of 2.9 s and a distance of 1.41 mm. Figure 3 shows the compressibility curves in the same way we can observe 3 axes that are force (N), time (s) and distance (mm), an arrow of the indicated point (Hardness) expresses the maximum force exerted to compress by 20% each of the bars, from which the parameters of distance and time used are obtained, all of these under the same initial conditions mentioned in the methodology of both tests. For example, in the T30 graph, it is observed that to compress the sample by 20%, a force of 118 N is required with a distance of 4 mm in 2.3 s.

### Organoleptic acceptability of the bars

The median age of the tasters was 20.5, with a range of 15 to 25 years old. The four types of bars with different concentrations of ant flour were evaluated. Taste, texture, and color were assessed, and they were expresses through a hedonic scale. The four types showed high acceptability, the percentage of the acceptability index was also high ( Table 4).

**Table 4.** Mean scores attributed to the energy bar formulations.

	Taste *		Texture *		Color *	
	Mean ± SD	AI (%)	Mean ± SD	AI (%)	Mean ± SD	AI (%)
T1	4.03 ± 0.18	95.7	3.84 ± 0.19	95.3	4.07 ± 0.17	95.8
T2	3.66 ± 0.23	94.1	3.74 ± 0.21	94.5	4.10 ± 0.17	96.1
T3	3.69 ± 0.24	93.7	3.84 ± 0.22	94.5	4.08 ± 0.19	95.5
T4	4.04 ± 0.18	95.6	3.84 ± 0.19	95.3	4.09 ± 0.18	95.8
Total	3.86 ± 0.13	97.3	3.81 ± 0.12	97.4	3.81 ± 0.12	97.9

Results are the mean of 100 evaluators. Acceptability Index (AI) = mean/upper limit*100.

* According to Kruskall-Wallis, p-values >0.05; therefore, the level of taste, texture, and color is the same in each of the formulations.

### Microbiological results

The analyzed sample was T4 for being the one with the highest amount of protein. The obtained values were within the limits allowed according to 071-MINSA-DIGESA-V01 (the sanitary technical norm in Peru) ( Table 5). ^28^

**Table 5.** Microorganism count in the energy bar formulations.

Microorganism	Permisible count *	Count	Unit
Mold	10 ^5^ × gr	1.0 × 10 ^1^	CFU/g
Salmonella	Absence	Absence	A-P/25g
Aerobic mesophilic microorganisms	1.0 × 10 ^4^ CFU/g	4.0 × 10 ^1^	CFU/g
Escherichia coli	10 MPN/g	<3	MPN/g
Yeast	10 CFU/g	1.0 × 10 ^1^	CFU/g

ICMSF Microbiological analysis.

* Based on 071-MINSA-DIGESA-V01. ^28^

## Discussion

The four types of bars with different percentages of ant flour were assessed (F1: 13%; F2: 17%; F3: 20%; F4: 23%). We used cereal such as quinoa and kiwicha, dried fruit (chestnuts, nuts, peanuts, almonds, and raisins). The proximate analysis showed that fat and proteins increased as the amount of ant flour increased as well, in comparison with the control bar made without ant flour (F0). Compressibility values ranged from 80.5 N to 117.6 N, and fracturability ranged from 8.48 to 12.33. We did not find any differences among the cereal formulations. All of them showed an acceptability index higher than 95%.

The fat and proteins increase with the addition of the ant flour is noticeable. A study with ant meal from the Rioja area in Peru showed 35.40% fat, 35.5% protein, with a pepsin digestibility of 99.77%. ^29^ It is known that the ants’ abdomen is fatty and that roasted ants are mixed with rice and cassava flour in Brazil and consumed regularly. ^30^ The protein value of insects has a composition similar to vertebrates’ such as pork, chicken, and fish. ^31^ Cephalotes ants have 42.59% protein, chicken 23%, and beef 20%. In addition, insects are high in sodium, potassium, zinc, magnesium, iron, copper, and calcium. ^14^^,^^32^ Other cereal bars made with similar products provide a lower amount of protein per serving.

In our study we obtained compressibility values ranging from 98.9 N to 117.6 N, which were obtained in the sample with the highest amount of protein (10.5 g). Alvarez obtained values from 130 N to 167 N in his cricket flour bars’ formulation, which was higher compared to our results. Alvarez attributed the difference to the protein content of the flour studied. However, it was concluded that the trend does not show significant differences due to the variability of the bars’ pieces, in addition to the structure of the bar because of the random distribution of dried fruit fragments. ^33^ A study with cereal bars with quinoa flakes reported values between 112 N and 216 N, ^18^ in which the sample with the highest hardness (216 N) that did not have quinoa in its composition was rated in the sensory analysis with “I slightly dislike it” and was associated with the hardness of the product as one of its characteristics. Some products with high fiber content are denser and harder; this does not imply a lower acceptance of the product. ^22^ but a bar easy to crumble and with excessively firmness would not be attractive to the consumer. At sensory level, this analysis correlates with the maximum force exerted between the molars of each panelist. ^34^

The fracturability test imitate chewing with the incisors. One study compared instrumental sensory texture to the maximum force exerted when biting (shear force), that research concluded that a bar with a higher breaking force might be crumbly but a bar that is easy to crumble does not imply that it requires a higher breaking force. ^34^ In our study, we observed a higher force required by the 25% and 30% treatments, and a lower force by the 15% and 20% treatments. The values obtained varied between 8.48 N to 12.33 N of compression. Marquez L obtained data between 23.9 to 33.8 N, which shows the wide range of firmness in these products when comparing this data with previous research. ^22^ According to a study on the addition of chontaduro flour to cereal bars, ^35^it is expected to observe different resistance characteristics given by the addition of different concentrations of the ingredients during the elaboration of the bars. Texture properties are linked to the composition of each ingredient in the product such as flour granulometry, structure, and nutritional value, which are manifested in the results obtained by the shear or compressive strength of the sample, which give a particular attribute to the product.

The four types of bars with different concentrations of ant flour were evaluated by 100 students. Taste, texture and color were evaluated and expressed by a hedonic scale. The acceptability index had values above 95 %. Although no other bars with *Atta sexdens* flour addition have been found to compare with, information has been collected from several cereal bars with different cereals in their composition. One study revealed that appearance is the limiting factor for consumer acceptability, essentially for cereal bars with dried fruits as ingredients. ^36^ They found higher acceptability when the bars had medium sugar concentration and high dietary fiber and β-glucan content. ^37^ In a cereal bar made with textured soy protein, wheat germ, and oats, enriched with ascorbic acid and α-tocopherol acetate. The formulation de Freitas evaluated was high in protein (15.31%), vitamin E (118.0 mg/100 g). The formulation with 1.1 g/100 g of ascorbic acid added obtained a higher significant sensory preference. ^38^

The results in this study showed absence of *Salmonella*, the count of molds and yeasts was 1.0 × 10 ^1^ CFU/g, 4.0 × 10 ^1^ CFU/g for aerobic mesophiles, and for *Escherichia coli* the result was <3 NMP/g. In general, the results were favorable and within the allowed limits. The cereal bars have low water activity, comply with sanitary specifications, and can be stored for 60 days. The yeasts, *Bacillus cereus* and fecal coliforms’ microbiological validation, performed by Gutkoski was acceptable for the Brazilian standards. ^37^

## Conclusions

The energy bars with ant flour were successfully developed and the sensory result of taste, texture and color attributes showed great acceptance, and any of them can be used for industrial scaling, especially the formulation containing 23% of ant flour that reached a high amount of protein. This product can be a good source of protein, fats and minerals and an alternative for consumers in general. The microbiological results of the energy bars were within the allowed parameters, making this product safe and suitable for consumption.

## Data Availability

Figshare: Risk perception. DOI: 10.6084/m9.figshare.23157566 Data are available under the terms of the Creative Commons Attribution 4.0 International license (CC-BY 4.0).
