# Supplementary material for: Development and acceptability of a cereal bar with Atta sexdens ant flour
Source: F1000Res. 2023 Jul 19;12:849. [Version 1] doi: 10.12688/f1000research.135516.1 (PMC11929954; doi:10.12688/f1000research.135516.1)
Supplement: Peer review history

# Reviewer report: Reviewer response for version 1

**DOI:** 10.5256/f1000research.148639.r309763

**Published:** 2024-09-13

**Authors:** Zabentungwa Hlongwane

**Affiliations:** 1 University of KwaZulu-Natal, Durban, South Africa

Title: The title needs to be improved and show that the nutritional composition of cereal bars enriched with ant meal was investigated

 Abstract: The abstract is good but it lacks a concluding statement what are the implications of the study findings?

 Keywords: “insect edible” confusing rephrase to edible insects

 Introduction

 Paragraph 1 First sentence: Change recommends to recommended

 Paragraph 1 second sentence: remove capital letter on It

 Paragraph 1 second sentence: No edible insects do not replace meat, they are sustainable, cheap, and rich in nutrients and can be used to combat malnutrition

 Paragraph 1 third sentence: “these insects” Which insects are you referring to because no insects were listed before this statement. You must mention the insects that are commonly consumed in the regions that you mentioned because they differ

 Paragraph 2 last sentence: Atta should be in italics

 Paragraph 3 second sentence: remove capital letter on In

 Paragraph 3 last sentence: It would be beneficial to explain why microbiological analysis and consumer acceptability were performed  

 Materials and methods

 The nutrient analysis is not very descriptive, were the samples measured in triplicates or duplicates? Which nitrogen analyzer did the use, and which procedure was used to determine the fat content?

 Consumer acceptability test

 The methods used are brief and not descriptive for example when were the panelists recruited, where was the study conducted, and where were the panelists seated? How much was the spacing between them? How was the food product presented which labels were used to differentiate between the treatments? Were the panelists selected randomly?  What was the ratio of males and females?

 Did the author use 9-point hedonic or 5-point hedonic scales and why?

 How did you ensure that the panelists were not allergic to edible ants?

 The panelists only focused on taste. The texture, aroma, and colour were not tested and why?

 How was the test environment and how did you ensure objectivity in the sensory evaluation method?

 Results

 The results are not well explained for example the description of results presented in table 2 only focused on protein and fat. What about Ash and carbohydrates? The author needs to say which treatment had the highest nutrients.

 Discussion

 In general, the author did not discuss the implications of his/her findings and hardly compared them to the work done by other scholars. In addition, the author needs to explain the cause and effect of his/her findings. The grammar can be improved in the discussion.

 Paragraph 1: “The proximate analysis showed that fat and proteins increased as the amount of ant flour increased as well, in comparison with the control bar made without ant flour (F0)” compare your findings with the work of other scholars and explain the factors that might have led to the increase in protein and fat content of energy bars.

 Paragraph 1: “Compressibility values ranged from 80.5 N to 117.6 N, and fracturability ranged from 8.48 to 12.33. We did not find any differences among the cereal formulations. All of them showed an acceptability index higher than 95%”  compare your findings with the findings of other scholars and explain cause and effect

 Paragraph 2: “ A study with ant meal from the Rioja area in Peru showed 35.40% fat, 35.5% protein” It is not clear what was investigated by this mentioned study, in addition, did the fat and protein content increase with the increasing percentage of ant meal added?

 Paragraph 2: “It is known that the ants’ abdomen is fatty and that roasted ants are mixed with rice and cassava flour in Brazil and consumed regularly” how is this relevant in this study?

 Paragraph 2: “The protein value of insects has a composition similar to vertebrates’ such as pork, chicken, and fish.31 Cephalotes ants have 42.59% protein, chicken 23%, and beef 20%. In addition, insects are high in sodium, potassium, zinc, magnesium, iron, copper, and calcium” How is this relevant to the findings of this study?

  Paragraph 2: Other cereal bars made with similar products provide a lower amount of protein per serving.” Which similar products? Why did you observe higher protein content while other studies observed lower protein content?

 Paragraph 4: “One study compared instrumental sensory texture to the maximum force exerted when biting (shear force), that research concluded that a bar with a higher breaking force might be crumbly but a bar that is easy to crumble does not imply that it requires a higher breaking force” rephrase this sentence and find a better way of reporting this

 Paragraph 4: “Marquez L” who is this? Is this supposed to be a citation?

 Paragraph 4: “According to a study on the addition of chontaduro flour to cereal bars” Chontaduro is not an ant or insect I don’t think it is relevant to compare cereal bars fortified with fruit flour to cereal bars fortified with insect flour as plants and animals have different nutrients and digestability.

 Paragraph 4: ”It  is expected to observe different resistance characteristics given by the addition of different concentrations of the ingredients during the elaboration of the bars” I don’t think so

 Paragraph 5: “One study revealed” this is does not sound right. I suggest you say According to Bchir et al. (2018)…… or Bchir et al. (2018) reported that…..

 Paragraph 5: “They found higher acceptability when the bars had medium sugar concentration and high dietary fiber and β-glucan content” This does not make sense, in the previous sentence you said a study by Bschir et al., 2018 revealed  however here you are saying they found They found higher acceptability when the bars

 had medium sugar concentration and high dietary fiber and β-glucan content.……..but you are citing a different author (37).

 Paragraph 5: “The formulation de Freitas evaluated was high in protein (15.31%), vitamin E (118.0 mg/100 g). The formulation with 1.1 g/100 g of ascorbic acid added obtained a higher significant sensory preference” this is difficult to follow

 Paragraph 6: “The results in this study showed absence of Salmonella, the count of molds and yeasts was 1.0 _ 101 CFU/g, 4.0 _ 101 CFU/g for aerobic mesophiles, and for Escherichia coli the result was <3 NMP/g. You need to explain what your findings mean and what are the implications of this.

 Conclusion

 The conclusion is too brief

 Any recommendations from the study? What was the significance of this study?

Is the work clearly and accurately presented and does it cite the current literature?

Partly

If applicable, is the statistical analysis and its interpretation appropriate?

Partly

Are all the source data underlying the results available to ensure full reproducibility?

Partly

Is the study design appropriate and is the work technically sound?

Partly

Are the conclusions drawn adequately supported by the results?

No

Are sufficient details of methods and analysis provided to allow replication by others?

No

Reviewer Expertise:

entomophagy, and entomology.

I confirm that I have read this submission and believe that I have an appropriate level of expertise to confirm that it is of an acceptable scientific standard, however I have significant reservations, as outlined above.

# Response

**Published:** 2024-10-18

**Authors:** Michelle Lozada-Urbano

**Affiliations:** Lima, Universidad Privada Norbert Wiener, Lima, Santa Beatriz, Peru

Dear Reviewer

 Zabentungwa Hlongwane

 We appreciate all the comments, we feel that they have helped to improve the paper.

 ​​​​​​ **Title: The title needs improvement and should indicate that the nutritional composition of cereal bars enriched with ant flour was investigated.** The title could be: "Nutritional Composition and Acceptability Evaluation of Cereal Bars Enriched with Ant Flour (Atta sexdens)." I have included this in the document.

**Response: **The change was made.

****

**Abstract: The abstract is good, but it lacks a concluding statement. What are the implications of the study's findings?**

**Keywords:** "Edible insect" is confusing; it should be rephrased as "edible insects."

**Introduction**

**Paragraph 1:** First sentence: Change "recommends" to "recommended."

**Response:** The word was changed to recommended.

**Paragraph 1, second sentence:** Remove the capitalization in "It."

**Response:** The capitalization was removed.

**Paragraph 1, second sentence:** Edible insects do not replace meat; they are sustainable, economical, nutrient-rich, and can be used to combat malnutrition.

**Response:** This sentence was modified.

**Paragraph 1, third sentence:** "These insects" - which insects are you referring to? Because insects were not mentioned prior to this statement. You should mention the insects commonly consumed in the regions you referred to, as they differ.

**Paragraph 2, last sentence:** "Atta" should be in italics.

**Response:** The word Atta was italicized.

**Paragraph 3, second sentence:** Remove the capitalization in "In."

**Response:** The capitalization was removed.

**Paragraph 3, last sentence:** It would be beneficial to explain why microbiological analyses and consumer acceptability assessments were conducted.

**Response:** This paragraph was improved.

**Materials and Methods**

 The nutrient analysis is not very descriptive. Were the samples measured in triplicate or duplicate? Which nitrogen analyzer was used, and what procedure was employed to determine the fat content?

**Response:** Moisture was determined according to AOAC 922.06. Fat was determined according to AOAC 925.09, and extraction was performed using Soxhlet. Ash was determined according to AOAC 923.03. Protein was determined according to AOAC 984.13, using 6.25 as the nitrogen-to-protein conversion factor for all samples; this determination was based on Kjeldahl methodology using conventional analysis. Carbohydrates were calculated by subtracting the percentages of fat, moisture, protein, ash, and carbohydrates from 100%. Analyses were performed in triplicate.

**Consumer Acceptability Test**

 The methods used are brief and not descriptive. For example, when were the panelists recruited? Where was the study conducted, and where did the panelists sit? What was the spacing between them? How was the food product presented? What labels were used to differentiate between the treatments? Were the panelists randomly selected? What was the gender ratio?

**Response:** The recruitment for the analysis took place in August 2020, in a school environment, and the participants were organized in groups of 10 students who entered according to their arrival at the venue. The study was conducted in a ventilated area, with folders set up containing the samples. The samples were served at room temperature in disposable containers, and each participant was provided with a personal bottle of water. The distance between chairs was one meter, and the labels had three-digit codes assigned to each sample. The percentage of women was 46%.

**Did the author use 9-point or 5-point hedonic scales, and why?**

**Response:** We consider that both scales are essential for measuring consumer preferences and product acceptability. Some advantages of using the 9-point scale are that it allows for capturing subtle differences in consumer preferences compared to the 5-point scale (Wichchukit and O'Mahony, 2015). The 9-point scale also supports more complex statistical analyses that can yield deeper insights into consumer behavior (Wichchukit and O'Mahony, 2015). However, we believe that the number of options can overwhelm respondents, resulting in less reliable data ("How rating scales influence the reliability of responses, the extreme points, the midpoint, and the preferences of respondents").

 While the 9-point scales provide detailed information, the number of points can be complex and may hinder responses, suggesting that simpler scales could be more effective in certain contexts. Therefore, we decided to use the 5-point scale, as it may be more effective due to its simplicity.

**Reference:** Wichchukit S, O'Mahony M. The 9-point hedonic scale and hedonic ranking in food science: some reappraisals and alternatives. Journal of the Science of Food and Agriculture (2015). doi: 10.1002/JSFA.6993.

**How did you ensure that the panelists were not allergic to edible ants?**

**Response:** They were asked, and none of them had previously consumed any products containing ants.

 The panelists only focused on the flavor. The color were not evaluated. Why?

**Response:** All samples had exactly the same color.

 What was the testing environment like, and how was objectivity ensured in the sensory evaluation method?

**Response:** The study was conducted in a ventilated area, with folders set up containing the samples. The samples were served at room temperature in disposable containers, and each participant was provided with a personal bottle of water. The distance between chairs was one meter, and the labels had three-digit codes assigned to each sample.

**Results**

 The results are not well explained. For example, the description of the results presented in Table 2 only focused on protein and fat. What about ash and carbohydrates? The author needs to indicate which treatment had the highest nutrient levels.

**Response:** Regarding moisture content, no significant difference was found between the formulations. Moisture levels ranged from 17.6 g in formulation F0 to 19.39 g in F2. For carbohydrate content, values varied from 60.65 g in formulation F4 to 66.28 g in F0, with no significant difference found. Although variations in moisture and carbohydrate content were observed in both, these were not statistically significant, suggesting that the variation in ant flour content does not impact these values.

 The ash analysis revealed significant differences between the formulations. Ash levels ranged from 1.23 g in formulation F4 to 1.86 g in F0. This variability suggests that the mineral composition of the different formulations decreases as the percentage of ant flour increases, which could be associated with the reduction of inputs such as quinoa and kiwicha.

**Discussion**

 In general, the author did not discuss the implications of their findings and barely compared them to the work of other scholars. Additionally, the author needs to explain the cause-and-effect relationships of their findings. The grammar in the discussion could be improved.

**Paragraph 1:** “The proximal analysis showed that fat and protein increased as the amount of ant flour increased, compared to the control bar made without ant flour (F0).” Compare your findings with the work of other scholars and explain the factors that may have contributed to the increase in protein and fat content in the energy bars.

**Response:** According to authors such as Guan et al. and Kowalski et al., the incorporation of insect flours, including crickets and mealworms, in bread and cakes showed an increase in protein, minerals, and healthy fatty acids (Guan et al., 2024; Kowalski et al., 2022). This result is similar in our cereal bars, where the additional protein allows for greater moisture, making them softer.

 Q.H., Guan., S., Qian., L., Chen., X.C., Feng. 1. Enhancing the nutritional value of bread by the addition of insect powder: a novel class of food protein additives. Journal of insects as food and feed, (2024). doi: 10.1163/23524588-20230123

 Stanisław, Kowalski., Anna, Mikulec., Magdalena, Skotnicka., Barbara, Mickowska., Małgorzata, Makarewicz., Renata, Sabat., Anna, Wywrocka-Gurgul., Aleksandra, Mazurek. 2. Effect of the Addition of Edible Insect Flour from Yellow Mealworm (Tenebrio molitor) on the Sensory Acceptance, and the Physicochemical and Textural Properties of Sponge Cake. Polish Journal of Food and Nutrition Sciences, (2022). doi: 10.31883/pjfns/155405

**Paragraph 1:** “The compressibility values ranged from 80.5 N to 117.6 N, and the fracturability varied from 8.48 to 12.33. We found no differences between the cereal formulations. All showed an acceptability index above 95%.” Compare your findings with those of other scholars and explain the cause-and-effect relationships.

**Response:** The comparison was made in paragraphs 3 and 4.

**Paragraph 2:** “A study with ant flour from the Rioja region in Peru showed 35.40% fat and 35.5% protein.” It is unclear what was investigated in this mentioned study. Additionally, did the fat and protein content increase with the increasing percentage of added ant flour?

**Response:** The meaning of the sentence was clarified in the complete paragraph.

**Paragraph 2:** “It is known that the abdomen of ants is fatty and that toasted ants are mixed with rice and cassava flour in Brazil and are regularly consumed.” What relevance does this have in this study?

**Response:** This line was removed from the text due to its lack of relevance.

**Paragraph 2:** “The protein value of insects has a composition similar to that of vertebrates, such as pork, chicken, and fish. Cephalotes ants have 42.59% protein, chicken has 23%, and beef has 20%. Additionally, insects are rich in sodium, potassium, zinc, magnesium, iron, copper, and calcium.” What relevance does this have for the findings of this study?

**Response:** It is relevant as it presents a comparison between the protein content of ants and the animal meats we typically consume, such as beef or chicken.

**Paragraph 2:** “Other cereal bars made with similar products provide a lower amount of protein per serving.” What similar products? Why did you observe a higher protein content while other studies reported lower protein content?

**Response:** The comparison is made with the study by Guan, who also finds a high protein and fat content.

**Paragraph 4:** “A study compared instrumental sensory texture with the maximum force exerted when biting (cutting force); that research concluded that a bar with greater breaking force could be brittle, but a bar that is easy to crumble does not imply it requires a greater breaking force.” Reformulate this sentence and find a better way to convey this information.

**Response:** The idea in this paragraph was reformulated.

**Paragraph 4:** “Marquez L” – who is this? Is this supposed to be a citation?

**Response:** This author was correctly cited.

**Paragraph 4:** “According to a study on the addition of chontaduro flour to cereal bars.” Chontaduro is neither an ant nor an insect. I don’t believe it is relevant to compare cereal bars fortified with fruit flour to cereal bars fortified with insect flour, as plants and animals have different nutrients and digestibility.

**Response:** The chontaduro is the larva of the black palm weevil ( *Rhynchophorus palmarum*). The scientific name has been included in the text.

**Paragraph 4: **“Different resistance characteristics are expected to be observed due to the addition of different concentrations of ingredients during the preparation of the bars.” I don't think this is accurate.

**Response:** The resistances were observed according to the percentage of ant flour used.

**Paragraph 5:** “A study revealed” does not sound correct. I suggest saying “According to Bchir et al. (2018)...” or “Bchir et al. (2018) reported that…”

**Response:** This citation was improved as follows: A study by Bchir et al. (2018) revealed that appearance is the main limiting factor for consumer acceptability, especially in cereal bars that include dried fruits as ingredients.

**Paragraph 5:** “They found higher acceptability when the bars had a medium sugar concentration and high dietary fiber and β-glucan content.” This does not make sense. In the previous sentence, you said that a study by Bchir et al. (2018) revealed this, but here you are saying that they found higher acceptability with medium sugar concentration and high dietary fiber and β-glucan content, citing a different author (37).

**Response:** The author Gutkoski found higher acceptability when the bars had medium sugar concentration and high dietary fiber and β-glucan content.

**Paragraph 5:** “The formulation evaluated by Freitas was high in protein (15.31%) and vitamin E (118.0 mg/100 g). The formulation with 1.1 g/100 g of added ascorbic acid achieved a significantly higher sensory preference.” This is difficult to follow.

**Response:** In a study by Gumul, nut bars with the addition of edible insect flours were evaluated, and this addition reduced the perception of most sensory attributes of the bars compared to the standard bar.

**Paragraph 6:** “The results in this study showed the absence of Salmonella, the count of molds and yeasts was 1.0 × 10^1 CFU/g, 4.0 × 10^1 CFU/g for aerobic mesophiles, and for Escherichia coli the result was <3 NMP/g.” You need to explain what your findings mean and what their implications are.

**Response:** The explanation of the findings and their implications was improved.

**Conclusion:**

 The conclusion is too brief.

**Response:** More conclusions were added to the text.

 Are there recommendations from the study? What was the importance of this study?

**Response:** A section with some recommendations was added.

# Reviewer report: Reviewer response for version 1

**DOI:** 10.5256/f1000research.148639.r288948

**Published:** 2024-07-01

**Authors:** Samuel Adelani Babarinde

**Affiliations:** 1 Ladoke Akintola University of Technology, Ogbomosho, Oyo, Nigeria

**REVIEWER’S COMMENTS TO AUTHORS**

**MANUSCRIPT TITILE:** **Development** **and acceptability of a cereal bar with** ***Atta sexdens*** **ant flour**

**Title**

**Modification is suggested**

 Modify as follows:

 Development and acceptability of a cereal bar with leaf cuter ant, *Atta sexdens,* flour  

**Abstract**

**Ln 2-3**

 Recast as follows:….the regions the central jungle where a leaf cutter ant, *Atta sexdens*

**L** **n 4-5**

 Recast as Department of… District of… Province of ……

**Ln 7-8**

 13%, 17&, 20% and 23% w/w or v/v or what?

**Ln 8-9**

 Recast as follows:… Analyses on moisture, totl fat…. and organoleptic indices (taste, texture, and color) were performed.

**Introduction**

**Pg 3, Paragraph 2,Ln 2**

 Change ‘ *Acheta dometiscus’* to ‘crickets’. The isnect is not a grasshopper.

 Change ‘Cameroon’ to ‘sub -Saharan Africa’ and add the following reference and 6 (Tamesse et al.):

 Babarinde SA, Mvumi BM, Babarinde GO, Manditsera FA, Akande TO, Adepoju, AA. Insects in food and feed systems in sub-Saharan Africa: the untapped potentials. *International Journal of Tropical Insect Science. *2021 41 (3): 1923-1951. https://doi.org/10.1007/s42690-020-00305-6

**Pg. 3, Paragraph 3, Ln 3**

 …. ‘similar quantities as fish’ or … ‘higher quntities than fish’

**Pg. 3, Paragraph 3, Ln 4**

 Change ‘protein from fish’ to ‘relying on insect protein.

**Pg. 3, Paragraph 4, Ln 2**

 Recast as follows: Cereals bars are in…….

**Pg 3, Paragraph 4, Ln 2**

 Insert ‘and’ before ’compression’

**Methods**

**Pg. 3, Paragraph 1**

*‘* Obtaining *Atta sexdens* ant’ should be changed to ‘Obtainingleaf cuter ant, *Atta sexdens’*

 The first paragraph is not part of what you did. It only provides information about the studied organism ( *Atta sexdens)* . Therefore, the content is better placed in the appropriate section of your Introduction.

**Pg. 3, Paragraph 2**

 Recast as Department of… District of… Province of ……

 ‘This was taken to an electric grinder …..’ should change to ‘This was milled with an electric grinder …..’

**Pg 3, Paragraph 5**

 Merge the paragraph with the preceding one.

**General Comments**

 You have to indicate how you formulated your Ant bar mix flour.

**Pg. 3, Paragraph 6, Ln 3**

 ..in different concentrations… You need to provide appropriate references.

**Figure 1**

 Change ‘Ceral’ to ‘Cereal’ in the figure legend: Figure 1: Cereal bar…….

**Pg. 5 Paragraph 1**

 Which hedonic scale did you use 1-9 or which? The, provide a brief description of what each number stands for.

**Pg. 5 Paragraph 2**

 Change ‘data was’ to ‘data were’. Data is plural, while datum is singular

**Results**

**Table 1**

 Why did you not compare the energy bar with ant flour as FO in Table 1? I feel this is necessary.

**Table 2**

 The methodology that led to your Table 2 is not quite explicit. You would need to improve the appropriate section of your methodology to allow your readers to understand what you did that led to the collection of the data in Table 2.

**Table 3**

 Indicate what F1-F4 stand for as footnote.

**Figure 2**

 Indicate what T1-T2 stand for as figure legend.

**Pg. 6, Paragraph 2**

 Did you use 13, 17, 20 and 23% or 15, 20, 25 and 30% ant meal?

**Figure 3**

 Indicate what T15-T30 stand for as figure legend.

 I am of the opinion that you shoud compare your treatments (T1-T4) with the cereal bar without and flour (F0).

**References**

 Add this to your references:

 (Babarinde et al., 2021)(Ref-1)

 Abstract

 Abstract has minor corrections highlighted in my Reviewer's comments to the Authors.

 Introduction

 The Introduction also has minor editorial corrections and the need to add more recent references.

 Methods

 There are few inconsistencies in the methods: for instance, the proportions of ant meal used for the experiment should be clarified.

 Discussion

 I feel the cereal bar without ant meal should be used as a standard to establish the results of the experiment.

Is the work clearly and accurately presented and does it cite the current literature?

Partly

If applicable, is the statistical analysis and its interpretation appropriate?

Yes

Are all the source data underlying the results available to ensure full reproducibility?

Yes

Is the study design appropriate and is the work technically sound?

Partly

Are the conclusions drawn adequately supported by the results?

Partly

Are sufficient details of methods and analysis provided to allow replication by others?

Yes

Reviewer Expertise:

Entomology and entomophagy

I confirm that I have read this submission and believe that I have an appropriate level of expertise to confirm that it is of an acceptable scientific standard, however I have significant reservations, as outlined above.

## References

[1] : Insects in food and feed systems in sub-Saharan Africa: the untapped potentials. *International Journal of Tropical Insect Science* .2021;41(3) : 10.1007/s42690-020-00305-6 1923-1951 10.1007/s42690-020-00305-6

# Response

**Published:** 2024-10-14

**Authors:** Michelle Lozada-Urbano

**Affiliations:** Lima, Universidad Privada Norbert Wiener, Lima, Santa Beatriz, Peru

Dear Reviewer, we appreciate your recommendations for change, each of them has been consistent and the changes have been made in the new document, including the new bibliography.
